# A Role for Maternal Factors in Suppressing Cytoplasmic Incompatibility

**DOI:** 10.3389/fmicb.2020.576844

**Published:** 2020-11-09

**Authors:** AJM Zehadee Momtaz, Abraham D. Ahumada Sabagh, Julian G. Gonzalez Amortegui, Samuel A. Salazar, Andrea Finessi, Jethel Hernandez, Steen Christensen, Laura R. Serbus

**Affiliations:** ^1^Department of Biological Sciences, Florida International University, Miami, FL, United States; ^2^Biomolecular Sciences Institute, Florida International University, Miami, FL, United States

**Keywords:** *Wolbachia*, cytoplasmic incompatibility, CI, rescue, *Drosophila*

## Abstract

*Wolbachia* are maternally transmitted bacterial endosymbionts, carried by approximately half of all insect species. *Wolbachia* prevalence in nature stems from manipulation of host reproduction to favor the success of infected females. The best known reproductive modification induced by *Wolbachia* is referred to as sperm-egg Cytoplasmic Incompatibility (CI). In CI, the sperm of *Wolbachia*-infected males cause embryonic lethality, attributed to paternal chromatin segregation defects during early mitotic divisions. Remarkably, the embryos of *Wolbachia-*infected females “rescue” CI lethality, yielding egg hatch rates equivalent to uninfected female crosses. Several models have been discussed as the basis for Rescue, and functional evidence indicates a major contribution by *Wolbachia* CI factors. A role for host contributions to Rescue remains largely untested. In this study, we used a chemical feeding approach to test for CI suppression capabilities by *Drosophila simulans*. We found that uninfected females exhibited significantly higher CI egg hatch rates in response to seven chemical treatments that affect DNA integrity, cell cycle control, and protein turnover. Three of these treatments suppressed CI induced by endogenous *w*Ri *Wolbachia*, as well as an ectopic *w*Mel *Wolbachia* infection. The results implicate DNA integrity as a focal aspect of CI suppression for different *Wolbachia* strains. The framework presented here, applied to diverse CI models, will further enrich our understanding of host reproductive manipulation by insect endosymbionts.

## Introduction

Endosymbiosis is a specialized form of interaction, with one organism dwelling inside the cells and tissues of another (Archibald, 2015). The bacterium *Wolbachia pipientis* is one of the most widespread endosymbionts, carried by half or more of all insect species (Weinert et al., 2015; Sazama et al., 2017). *Wolbachia* are gram negative bacteria that belong to the alpha-protobacterial class *Rickettsiales*. *Wolbachia* are maternally transmitted, with the efficacy of transmission dependent on the bacteria being loaded into eggs (Breeuwer and Werren, 1990; Zchori-Fein et al., 1998; Hadfield and Axton, 1999; Stouthamer et al., 1999; Rasgon and Scott, 2003; Veneti et al., 2004; Ferree et al., 2005; Serbus et al., 2008; Fast et al., 2011). *Wolbachia* commonly modify host reproduction to favor the success of infected females. This is accomplished by induction of parthenogenesis, male killing, feminization and sperm-egg cytoplasmic incompatibility (Yen and Barr, 1971; Rousset et al., 1992; Stouthamer et al., 1993; Jiggins et al., 1998; Hurst et al., 1999; Werren et al., 2008). Though *Wolbachia* interactions with their host appear generally commensal, this extent of host manipulation classifies *Wolbachia* bacteria as reproductive parasites.

Cytoplasmic incompatibility (CI) is the most widely known of all *Wolbachia*-induced reproductive manipulations (Hoffmann and Turelli, 1997; Werren, 1997; Stouthamer et al., 1999). CI is characterized by embryonic lethality in crosses between uninfected females and *Wolbachia*-infected males (Hertig, 1936; Laven, 1959; Yen and Barr, 1971, 1973; Noda, 1984; Hsiao and Hsiao, 1985; Wade and Stevens, 1985; Hoffmann et al., 1986; Binnington and Hoffmann, 1989). By contrast, *Wolbachia*-infected females are compatible with both uninfected and *Wolbachia*-infected males, with viable progeny produced by both types of crosses. The ability of embryos from *Wolbachia*-infected females to survive the *Wolbachia*-modified sperm is known as “Rescue.” Compatibility is conferred by specific pairings of sperm modification (mod) and rescue capacity (resc) associated with different *Wolbachia* strains (Turelli, 1994; Werren, 1997; McGraw and O’Neill, 1999; Charlat et al., 2001; Poinsot et al., 2003; Duron et al., 2006; Zabalou et al., 2008; Nor et al., 2013). With infected females favored by elimination of incompatible embryos, the CI/Rescue paradigm effectively drives host population replacement in natural populations as well as in applied, vector management scenarios (Turelli and Hoffmann, 1991, 1995; Riegler et al., 2005; Hoffmann et al., 2011; Kriesner et al., 2013; Schuler et al., 2013; Schmidt et al., 2017; Turelli et al., 2018).

The cellular basis of CI has been a point of interest for many years. Cytological experiments indicate that mitotic defects are a consensus feature of CI across *Wolbachia*-host systems. Specifically, paternal chromatin remains at the metaphase plate while maternal chromatin segregates to opposite poles in anaphase, resulting in chromosome bridging and aneuploidy (O’Neill and Karr, 1990; Reed and Werren, 1995; Callaini and Riparbelli, 1996; Lassy and Karr, 1996; Callaini et al., 1997; Tram and Sullivan, 2002; Landmann et al., 2009; Bonneau et al., 2018b). Studies from *Nasonia* and *Drosophila simulans* have suggested these mitotic defects are produced from a timing mismatch between male and female pronuclei at the first mitotic division, which must be reconciled in order to enable Rescue (Callaini et al., 1997; Tram and Sullivan, 2002; Landmann et al., 2009). A separate line of work implicated *Wolbachia-*induced oxidative damage to spermatocyte DNA as a contributor to CI lethality (Brennan et al., 2012) with the implication that DNA damage prevention and/or repair in the embryo is important in conferring Rescue. This model is consistent with a body of literature on *Wolbachia* and induction of oxidative stress (Brennan et al., 2008; Xi et al., 2008; Hughes et al., 2011; Andrews et al., 2012; Pan et al., 2012; Bian et al., 2013; Zug and Hammerstein, 2015).

The most recent model is that *Wolbachia* CI factors (Cifs) are responsible for both CI and Rescue (Beckmann et al., 2017; LePage et al., 2017; Shropshire et al., 2018). Bioinformatic predictions and transgenic studies using yeast and *Drosophila melanogaster* models, have indicated that CifB proteins have deubiquitlase activity (CidB), nuclease activity (CinB), or both (CndB) (Beckmann and Fallon, 2013; Beckmann et al., 2017, 2019a,b; Lindsey et al., 2018; Chen et al., 2019). CifB is required to induce CI (LePage et al., 2017; Bonneau et al., 2018b, 2019; Chen et al., 2019; Meany et al., 2019), possibly by affecting sperm chromatin remodeling during spermatogenesis (Beckmann et al., 2019b). By contrast, CifA can Rescue classical CI phenotypes induced by *Wolbachia* (Shropshire et al., 2018), as well as CI associated with dual expression of CifA and CifB in transgenic males (Chen et al., 2019; Shropshire and Bordenstein, 2019). Proteomic evidence also indicates that CidA binding modifies CidB targeting (Beckmann et al., 2019b).

One caveat of the CifA findings to date is that *D. melanogaster* exhibits transient CI, mainly in association with newly eclosed males (Reynolds and Hoffmann, 2002; Yamada et al., 2007; LePage et al., 2017), particularly for first-emerging males of a population (Yamada et al., 2007). Depending upon the host strain used, initial hatch rates of 5–50% increase to 50–80% by day 3, and become normal by day 5 (Reynolds and Hoffmann, 2002; Yamada et al., 2007; LePage et al., 2017). This CI decline appears attributable to the host background, as *w*Ri *Wolbachia* transinfected into *D. melanogaster* also elicit a mild CI response, allowing on the order of 70% egg hatch (Boyle et al., 1993). By contrast, progeny produced by *w*Ri *Wolbachia* CI range from 0 to 6% of normal for newly eclosed males, 15–30% in 7–9 day old males, and 40–50% in 14-day old males, depending upon the experiment (Hoffmann et al., 1986; Turelli and Hoffmann, 1995). Thus, it is unclear if *D. melanogaster* CI represents mild induction of the CI defect, a background environment that is already highly permissive/enabling of Rescue, or both. As transgenic studies of Cif function in CI and Rescue have only just begun, it is not known to what extent Cif-related mechanisms in *D. melanogaster* represent that of other hosts exhibiting severe, *Wolbachia-*induced CI defects.

A long-standing question has been to what extent host factors contribute to the mechanism of Rescue. If Cif proteins act exclusively in terms of a toxin-antitoxin system, with CifA suppressing CifB function via direct binding (Beckmann et al., 2019a), no host involvement is required for Rescue. Consistent with the toxin–antitoxin model, direct binding has been demonstrated for multiple cognate pairs of CifA and CifB proteins (Beckmann et al., 2017, 2019b; Chen et al., 2019). Complementary studies did not identify contributions by additional *Wolbachia-*generated factors (Bonneau et al., 2018a, 2019; Perlmutter and Bordenstein, 2020), though host genetic background differences are reported to alter CI severity (Boyle et al., 1993; Poinsot et al., 1998). It remains possible that host mechanisms, to some extent, run in support of, in parallel to, or independently of bacterial effectors in the context of Rescue. *Cardinium* endosymbionts carried by *Encarsia* wasps have been credited with inducing their own forms of CI and Rescue (Hunter et al., 2003; Penz et al., 2012; Mann et al., 2017), in the apparent absence of Cif proteins altogether (Lindsey et al., 2018; Doremus and Hunter, 2020). Two additional non-*Wolbachia* CI systems were recently identified (Doremus and Hunter, 2020), in *Lariophagus* wasps (König et al., 2019) as well as in *Brontispa* beetles (Takano et al., 2017). While it is possible that each endosymbiont has a self-contained mechanism for CI and Rescue, another possibility is that these functionally convergent phenotypes are due to endosymbiont effects on conserved, cellular processes of the host. As such, the extent of host involvement in the process of Rescue merits examination.

Since Rescue involves preventing and/or repairing CI defects, this study examined whether *D. simulans* females have the capacity to alter developmental outcomes of CI embryos. If this is possible, then modifying the function of the relevant host pathways should confer CI suppression, evident as increased hatch rates for uninfected embryos that are otherwise subject to CI lethality. To test this, uninfected *D. simulans* females were exposed to chemicals that alter candidate cellular processes, previously implicated in CI and Rescue. CI induced by the endogenous *w*Ri *Wolbachia* strain, as well as a transinfected *w*Mel *Wolbachia* strain, were investigated. Egg hatch data were evaluated in light of existing CI/Rescue models, as described below.

## Materials and Methods

### Fly Stocks and Rearing Conditions

The *w*Ri *Wolbachia* strain, endogenous to *D. simulans*, used in this study was originally described by Hoffman, Turrelli, and Simmons (Hoffmann et al., 1986). The uninfected *D. simulans* strain (w^–^) is of the same genetic background, as it was this original line cured of *Wolbachia* with tetracycline. The *w*Mel trans-infected line was created with *Wolbachia* from *D. melanogaster* (Poinsot et al., 1998), backcrossed into the cured fly stock for six generations to standardize the *D. simulans* genetic background. We previously confirmed the identity of *w*Ri and *w*Mel in *D. simulans*, and verified that the *w*Mel transinfection matches the standard *w*Mel strain carried by most *D. melanogaster* stocks (Christensen et al., 2016).

All the flies used in this study were maintained at 25°C on a 12 h light/dark cycle using an Invictus Drosophila incubator (Genessee Scientific, United States). Flies were raised in standard 6oz square bottom polyethylene bottles containing 25–30 ml of fly food (described later). Each stock bottle was seeded by approximately 80–100 flies, a mixture of both male and female, and incubated for 3–6 days. After this period, flies were either transferred to a new bottle or discarded. Flies used to seed all bottles were discarded by 12–15 days of age. To collect virgin flies, stock bottles were completely cleared, and rechecked by eye to verify the absence of flies. Newly eclosed flies were collected 5–8 h later using standard CO_2_ gas pads. Males and females were separated and temporarily stored in narrow polypropylene vials until loading into treatment vials/plates, as described below. To avoid damage from prolonged exposure to CO_2_, fly sorting was limited to a 20–25 min time frame.

### Microbial 16S rRNA Gene Sequencing

To determine the infection status in *D. simulans* flies, microbial 16S rRNA gene sequencing was performed. Ovaries were dissected from uninfected, *w*Ri-infected and *w*Mel-infected *D. simulans* females, followed by sequencing of region V1–V3 of the 16S rRNA gene, carried out on an Illumina MiSeq as previously described (Christensen et al., 2019). Each sample represents ovarian content from 20 flies. The raw sequencing data are available at https://www.ncbi.nlm.nih.gov/Traces/study/?acc=PRJNA663645.

### Food Preparation

The stock food used in this study was made as per a standard Bloomington Stock Center recipe, described earlier^1^ (Camacho et al., 2017). For chemical feeding assays, concentrated stock solutions of each chemical were prepared using an appropriate solvent, dependent on necessary final concentration. To make chemical food for independent experiments, the appropriate amount of stock solution was mixed with melted food and mixed thoroughly by stirring. This food was transferred to either vials or plate wells immediately, before cooling and solidifying. The same amount of solvent was mixed with standard food to create a parallel “control food” condition in each experiment. Vial-based trials contained 3–5 ml of food per vial, while in the 24-well plate format, each well contained 800 μL of food.

### Dose Response Curve Preparation

To determine the appropriate feeding concentration for each chemical, a range of 6–7 doses was empirically tested for each compound. The range of concentrations used was based on information available in existing literature. Each chemical was diluted to the appropriate concentration in beakers containing 10 mL standard food with Brilliant Blue G food added (Acros Organics). All content was mixed thoroughly for 30 s, then divided equally into two treatment vials and cooled under the fume hood for ∼2 h to prevent condensation from collecting along the sides of the vials. In each case, one treatment vial was immediately used, and the second vial was plugged with rayon, wrapped in foil, and stored in a sealed container at 4°C for use 6 days later.

To carry out dose-response testing, six uninfected male and six uninfected female *D. simulans* flies were incubated in the first set of treatment vials. On the 6^*th*^ day, the flies were transferred to the second corresponding treatment vials for an additional 6 days. Adult mortality, egg lay, egg hatch and larval development were qualitatively scored for treatment vials and corresponding controls across the 12-day period. Treatment vials equivalent to the control were scored as “+.” Conditions exhibiting loss of 40% or more relative to the control, of eggs, larvae and/or pupae, were separately noted. Defects occurring during later days (Zchori-Fein et al., 1998; Rasgon and Scott, 2003; Veneti et al., 2004; Ferree et al., 2005; Serbus et al., 2008; Fast et al., 2011) of treatment were scored as “some,” and consistent developmental defects across the 12-day span were scored as “–.” The highest concentration of chemical with no adverse effect on flies as per the above criteria was selected for subsequent feeding assays. Dose response curves for dual drug treatment combinations were carried out similarly. Two independent biological replicates were performed for all dose-response experiments.

### CI Suppression Tests

#### In the Vial-Based Format

Virgin *D. simulans* flies were incubated for 3 days in vials containing 3–5 ml of food. Females were split between treatment food and control food conditions, whereas infected males were exposed to standard food only. In all cases, flies were grouped, with 15–20 flies per vial. On day 3, male and female flies were transferred to fresh vials of standard food for an 8-h mating period. Depending upon the experiment, this was done as single pair matings or as mass matings of 30–40 flies, using equal numbers of males and females. Afterward, male flies were discarded and female flies were returned their original treatment vials. At day 4, individual females were split up into separate vials that sustained their existing treatment conditions, with the addition of blue food coloring to the food to improve egg visibility.

#### In the Plate Assay Format

A detailed description is provided in Additional File S1. Briefly, Corning 24-well plates (Cat# 3738) were set up with 800 μL of fly food per well. 10 *D. simulans* virgin females were added to each well and incubated for 3 days. Uninfected females were added to eight standard food wells for use as the CI control, and to eight treatment wells to test for chemical suppression of CI. Infected females were added to eight standard food wells for use as a Rescue control. *Wolbachia-*infected males were incubated separately on standard food for 3 days, at a density of 45 flies or fewer per vial. At day 3, the females were transferred to a new plate carrying standard food and mated to 10 infected males for 8 h. Afterward, males were removed, and female flies returned to their respective wells in the original treatment plate. At day 4, females were transferred to a fresh plate that contained the same treatment condition per well, along with blue food coloring.

#### For Both Assay Formats

Female flies were discarded at day 5, followed by scoring of egg hatch at day 6. Two or more independent biological replicates were performed for each plate assay experiment. For rigor and consistency across the plate assays, only plates that showed a 12% or lower hatch rate for the CI control were scored.

### Statistical Analysis

Chi Square tests of goodness of fit were performed manually as per standard procedures (McDonald, 2014). A Bonferonni correction was applied, so that alpha values were scaled to the number of data categories analyzed (McDonald, 2014). Z′ values were calculated as previously (Zhang et al., 1999; Serbus et al., 2012). As previously, the IBM SPSS v.23 analysis package was used for all other statistical test (Field, 2013; Christensen et al., 2019). The data were analyzed for normality using the Shapiro–Wilk test and for homogeneity of variance by Levene’s test (Shapiro and Wilk, 1965; Lim and Loh, 1996; Mohd Razali and Bee, 2011). For the data with normal distribution, mean differences were evaluated using a *t*-test if variance was homogeneous, and Welch’s *t*-test if it was not (McDonald, 2014; Rietveld and van Hout, 2015). If the data did not fit the normal distribution, the Mann–Whitney *U-*test was performed for data showing homogeneity of variance, and an independent *t*-test was performed with bootstrapping as an approximation when variance was uneven (LaFlair et al., 2015; Rietveld and van Hout, 2015). To assess the power of different sample sizes, we also used a bootstrap procedure in MATLAB^TM^ (Mathworks, Natick, MA, United States) that randomly sub-samples from the data to determine the sample size required to meet specified *p*-values (Christensen et al., 2019).

## Results

### Verification of *D. simulans* Endosymbiont Identity by 16S rRNA Analysis

The *D. simulans* flies used in this study carry the *Wolbachia* strain *w*Ri as a natural infection (Hoffmann et al., 1986), or *w*Mel as a transinfected strain (Poinsot et al., 1998). Female flies from these strains have been shown by DNA staining to exhibit nucleoids in their germline cells that are consistent with *Wolbachia* infection (Serbus and Sullivan, 2007; Christensen et al., 2016). These lines have also been confirmed as PCR-positive for the *Wolbachia surface protein* (*Wsp*) gene, and the *Wolbachia* strain identities have been confirmed by sequencing (Christensen et al., 2016). Use of these detection methods, while consistent with expected *Wolbachia* identities, does not rule out the possible presence of other bacterial endosymbionts.

**FIGURE 1:**
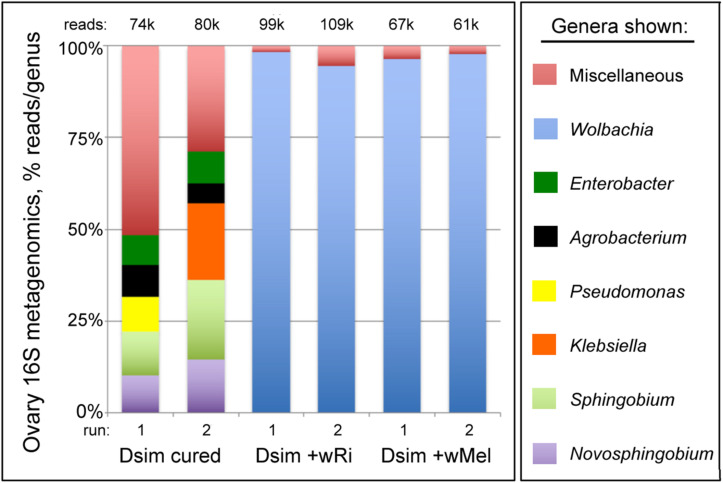
16S rRNA microbiome profiles associated with *Drosophila simulans* ovary tissues. Uninfected and *Wolbachia*-infected tissues are shown. Top five most abundant genera that equal or exceed 1% abundance per sample are shown. For further details, see Supplementary Table S1 and Additional File S2.

To independently confirm the identity of the germline bacteria carried by *Wolbachia*-infected flies, 16S rRNA microbiome analyses were carried out as previously described (Christensen et al., 2019). Ovary tissue samples were analyzed from both uninfected and *Wolbachia*-infected *D. simulans* lines. The data indicate *Wolbachia* spp. as the predominant taxon carried by both *w*Ri- and *w*Mel-infected tissues, with 94.5–98.3% of the reads representing the *Wolbachia* genus (Figure 1) (Supplementary Table S1) (Additional File S2). The other non-*Wolbachia* taxa detected in the *Wolbachia-*infected ovary samples paralleled that of the uninfected control (Figure 1). As this was a non-sterile assay, these signatures likely reflect the microbiome of the cuticle, the body cavity and residual contamination of dissection equipment (Christensen et al., 2019). Notably, the *Wolbachia-*infected samples show no evidence of other *Drosophila-*resident symbionts such as *Spiroplasma*. Thus, *Wolbachia* endosymbionts represent the vast majority, if not all, of the microbiome carried by maternal germline cells that generate Rescue-capable eggs.

### Confirmation of Baseline CI and Rescue Phenotypes From *D. simulans*

Previous studies have demonstrated that natural (*w*Ri) as well as transinfected (*w*Mel) *Wolbachia* strains induce robust CI and Rescue effects in *D. simulans* (Hoffmann et al., 1986; Binnington and Hoffmann, 1989; Poinsot et al., 1998). To determine the strength of CI and Rescue in current laboratory settings, group mating assays were performed and egg hatch outcomes were scored for individual females. Crosses of infected females to infected males, referred to as the Rescue, yielded a 92% hatch rate. This was not significantly different from the uninfected Control cross (*p* > 0.05; adjusted α = 0.0083) (Table 1). By contrast, the CI egg hatch rates ranged from 4 to 10% for *w*Mel and *w*Ri. This represents a significant reduction in hatch rates, as compared to both Control and Rescue crosses (*p* < 0.001; adjusted α = 0.0083) (Table 1). This outcome is consistent with the expectation of strong CI and Rescue phenotypes associated with *D. simulans*.

**TABLE 1:** Egg hatch rates from CI-related crosses on control food.

	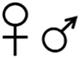		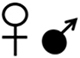		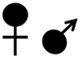	
Egg hatch rates on control food conditions	Control cross		CI cross		Rescue cross	
			
	Hatch rate	Eggs (females)	Hatch rate	Eggs (females)	Hatch rate	Eggs (females)
Mass matings: *D. simulans w*Ri	89%	606 (31)	10%	712 (41)	92%	575 (35)
Mass matings: *D. simulans w*Mel	89%	561 (41)	4%	546 (38)	92%	820 (42)
Single pair matings confirmed: *D. simulans w*Ri	90%	679 (35)	10%	672 (43)	n/d	n/d

*Wolbachia* infection has been reported to alter pheromone release and perception by other species of *Drosophila*, leading to altered mating patterns (Ringo et al., 2011; Schneider et al., 2019). Thus, it is formally possible that low egg hatch in incompatible crosses, normally attributed to *w*Ri-induced CI lethality, instead reflects a failure to mate. To distinguish between these possibilities, single pair matings were set up, with egg hatch scored only for vials in which mating was visually confirmed. In these experiments, hatch rates remained low for CI crosses (10%) as compared to Control crosses (90%) (Table 1) (*p* < 0.001; adjusted α = 0.0125). The results of these mating-confirmed crosses closely parallels that shown above for group matings. This demonstrates that the low hatch rates currently associated with *w*Ri-induced CI are due to the reduced viability of CI eggs laid by uninfected *D. simulans* females.

### Demonstrating Use of Small Molecule Inhibitors to Suppress CI Phenotypes

CI embryos have previously been shown to exhibit defective incorporation of maternal histones into paternal DNA (Landmann et al., 2009). We reasoned that chromatin-modifying compounds may also be able to confer CI suppression in a manner analogous to natural Rescue. It is known that acetylation of histones lowers their affinity for DNA and loosens chromatin structure, whereas removal of acetyl groups by histone de-acetylase (HDAC) enzymes reverses this effect (Pasyukova and Vaiserman, 2017). Since HDAC inhibitor compounds are commonly used in animal models and clinical settings (Drummond et al., 2005; Ganai et al., 2016; Tandon et al., 2016; Pasyukova and Vaiserman, 2017; Salcedo Magguilli, 2017), an array of well-established compounds is available for testing. Thus, we established a chemical feeding protocol to test a role for chromatin remodeling in CI suppression.

**FIGURE 2:**
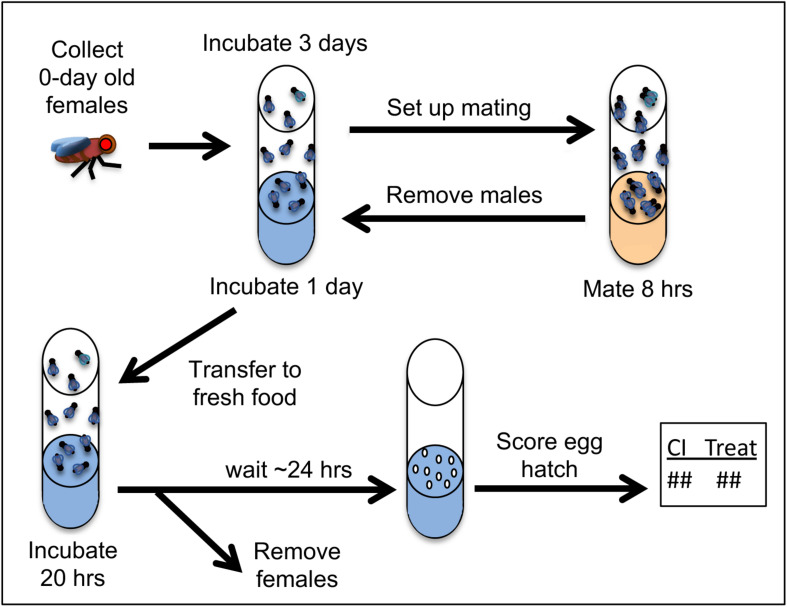
Procedures used for CI- and Rescue-related crosses. Drug feedings were carried out on blue food where specified in the protocol timeline. For details on food preparation, please see section “Materials and Methods” and Additional File S1.

One of the most well-known HDAC inhibitors is the non-toxic, short chain fatty acid butyric acid, or sodium butyrate (NaBu) (Cousens et al., 1979; Davie, 2003). Dose-response assays were performed to determine the maximum tolerable NaBu dosage for adult *D. simulans*. Doses within a 200-fold range were tested, and the highest dose which did not substantially alter developmental phenotypes was identified (Supplementary Table S2). This dose was used for all subsequent tests for NaBu impact on CI hatch rates. It is not known what stage(s) of oogenesis are important for conferring Rescue ability upon embryos. Thus, uninfected females were kept on drug food throughout the duration of the experiment except during matings with CI males. Males were incubated and mated on control food only to prevent ingestion of the compound (Figure 2).

Group mating experiments were performed to determine the impact of NaBu on CI hatch rates associated with *w*Ri and *w*Mel *Wolbachia*. For *w*Ri, the CI hatch rate was 1.6-fold higher for the NaBu treated vials when compared to control food (*p* = 0.0122; adjusted α = 0.0125) (Table 2). For *w*Mel, the CI hatch rate on NaBu food was more than twice that of control food conditions (*p* < 0.001 adjusted α = 0.0125). To further verify whether the increased egg hatch rates with NaBu feeding were due to unanticipated changes in the mating behavior, single pair matings were also performed. The data confirmed a 94% hatch rate for Rescue crosses, as compared to a 10% hatch rate for CI (*p* < 0.001; adjusted α = 0.0083). For the NaBu treatment condition, CI hatch rates were nearly double that of the CI control (*p* < 0.001; adjusted α = 0.0083) (Table 2). Taken together, these data indicate that the HDAC inhibitor NaBu induces CI suppression in uninfected females.

**TABLE 2:** Egg hatch rates from CI-related crosses on NaBu treatment food.

	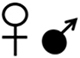		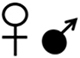		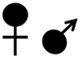	
Egg hatch rates in response to NaBu	CI cross Regular food		CI cross NaBu food		Rescue cross Regular food	
			
	Hatch rate	Eggs (females)	Hatch rate	Eggs (females)	Hatch rate	Eggs (females)
Mass matings: *D. simulans w*Ri	10%	454 (30)	16%	292 (21)	n/d	n/d
Mass matings: *D. simulans w*Mel	4%	382 (29)	11%	544 (40)	n/d	n/d
Single pair matings confirmed: *D. simulans w*Ri	10%	771 (30)	19%	724 (32)	94%	741 (30)

### Scaling Up Screening of Small Molecule Inhibitors to Test for CI Suppression

The observation that CI can be partially suppressed by NaBu raises potential questions of whether targeting other host factors could confer CI suppression as well. Assessing this possibility requires screening of more compounds, including sufficient replicates to distinguish CI suppression effects. To this end, we developed a plate-based feeding assay for analyzing small molecule effects on CI hatch rates, based upon existing adult screening methods (Markstein et al., 2014) that were previously optimized for use in *Wolbachia* assays (Christensen et al., 2019). In the context of 24-well plates, a maximum of 8 wells can be analyzed per condition for CI, CI+treatment and Rescue. Ensuring that results would be relevant to those of a naturally robust CI system, *w*Ri-infected males were used to perform all plate assay matings.

**FIGURE 3:**
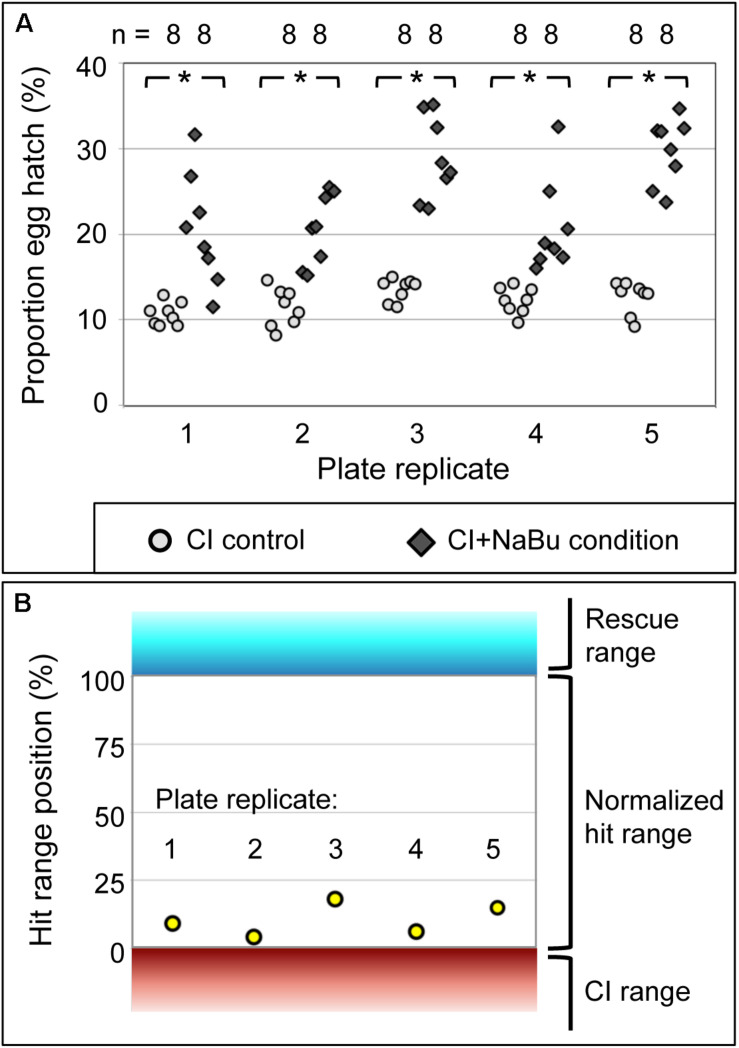
The impact of NaBu treatment on CI egg hatch. **(A)** Hatch rate data from assay plates using *w*Ri-infected *D. simulans* flies. Each symbol represents data from a single well. *p < 0.005. **(B)** NaBu impact on CI, in terms of conventional Z′ analysis. Range boundaries of the CI control (red) and Rescue control (cyan) are indicated. The normalized “hit range” between controls is shown in white. Yellow dots: average hatch rate for the CI+NaBu condition per screening plate, normalized to the range between CI and Rescue controls. For further details, see Supplementary Tables S4, S5.

To determine whether chemical feeding in a plate assay format is an effective means of identifying CI suppression, the HDAC inhibitor NaBu was retested across five biologically independent plate replicates. All plate replicates indicated consistently higher egg hatch for the CI+NaBu condition (20–30%) than was seen in the CI control condition (11–14%) (*p*-value range: <0.001 to 0.003) (Figure 3A) (Supplementary Tables S3, S4). It is also possible to consider the data from the perspective of plate-based cell screens, where the quality of such assays is typically described in terms of its Z′ factor. Positive Z′ values, ranging between 0 and 1, only result when the average values for the controls are separated by more than three times the standard deviation for each treatment (Zhang et al., 1999; Birmingham et al., 2009; Serbus et al., 2012). According to this analysis, data from the five NaBu plate replicates returned Z′ values ranging from 0.76 to 0.89 (Supplementary Table S5). The CI+NaBu condition also occupied the intermediate “hit” range, consistently distinguishable from the CI control (Figure 3B). This demonstrates that the plate-based feeding assay reproducibly identifies CI-suppressing treatments.

**FIGURE 4:**
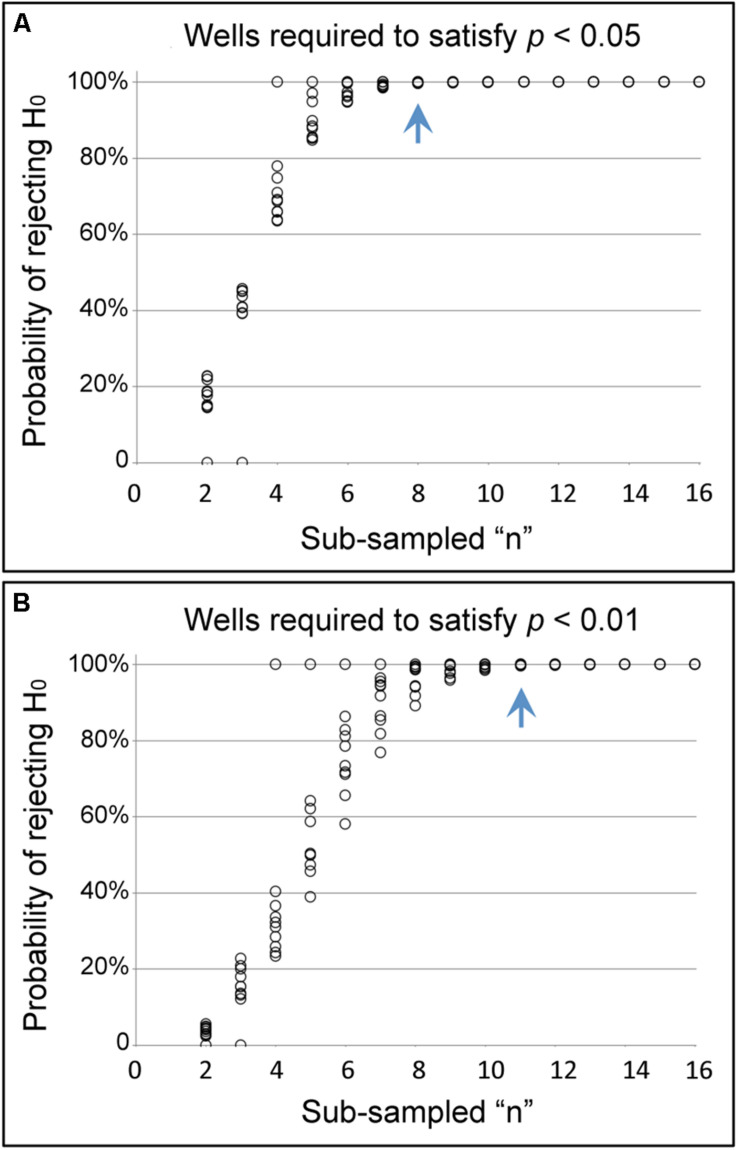
Identifying sufficient sample size in the pate assay format. The graphs show the likelihood of seeing a significant difference in hatch rate between CI and CI+NaBu conditions, as defined by **(A)** the conventional α-value of 0.05, as well as **(B)** the more stringent α-value of 0.01. The blue arrow indicates the number of wells at which the probability of rejecting the null hypothesis has reached 99.5% or higher for all sub-sampled datasets analyzed.

To determine the quantity of screening plates required for reproducible identification of a chemical suppressor of CI, the NaBu plate data were statistically analyzed. Data were collated and compared for every crosswise pairing of five independent screening plates, using data from 16 wells per condition in each case. Sub-sampling among the five plate replicates indicated that data combinations from any two plate replicates identified a significant difference between CI and CI+NaBu conditions (*p* < 0.001, *n* = 10 plate data combinations) (Supplementary Table S4). To further determine how many wells are required for significance, data were sub-sampled from within each of the paired plate datasets (Christensen et al., 2019). Data from eight or more wells per condition were sufficient to identify significant differences between CI and CI+NaBu conditions, when setting an alpha value at 0.05 (Figure 4A). Sampling from 11 or more wells per condition yielded a significant difference with alpha set at 0.01 (*n* = 10 plate data combinations) (Figure 4B). These data indicate that screening two chemical assay plates is sufficient to detect chemical suppressors of CI. In addition to corroborating NaBu-induced CI suppression, these plate assay data also created the foundation for testing additional candidate compounds.

### Testing for CI Suppression by Short-Chain Fatty Acids and Protein Acetylation Modifier

To further pursue the functional role of NaBu in CI suppression, the basic structure of this compound was considered. Since NaBu is a short-chain fatty acid, this opens the question of whether short-chain fatty acids generally exert CI-suppressing effects. To investigate this possibility, flies were fed with three other forms of short-chain fatty acids, specifically acetic, propionic and valeric acid, to test for suppression of *w*Ri-induced CI in *D. simulans.* The doses used for each compound, as well as all others described below, were empirically determined in vial format (Supplementary Table S6), then tested for impact on CI hatch rates in the context of the plate-based assay. Results from these experiments indicated that acetic acid conferred borderline CI suppression abilities upon uninfected embryos (*p* = 0.047). Propionic acid and valeric acid had no significant effect on CI hatch rates (Table 3) (Supplementary Tables S7, S8). Thus, CI suppression is not a generalized effect associated with dietary short-chain fatty acids.

**TABLE 3:** Impact of chemical treatments on CI egg hatch rates for *w*Ri-infected *D. simulans.*

Effect tested	Chemical used	Reputed cellular effect	Dose used	# wells (plates)	Significant increase in hatch rate?	*p*-value
Diverse functions	NaBu	C4 short chain fatty acid. Affects HDACs, DNA damage repair, cell cycle	50 mM	16 (2)	Yes	< 0.001
Short chain fatty acids	Acetic acid	C2 short chain fatty acid	100 mM	16 (2)	Borderline	0.047
	Propionic acid	C3 short chain fatty acid	10 mM	16 (2)	No	0.769
	Valeric acid	C5 short chain fatty acid	5 mM	16 (2)	No	0.926
Chromatin modification	Quisinostat	HDAC inhibitor	1 uM	16 (2)	No	0.235
	Trichostatin A	HDAC inhibitor	10 uM	16 (2)	No	0.696
	Vorinostat/SAHA	HDAC inhibitor	75 uM	16 (2)	No	0.913
	CUDC-101	Inhibits HDACs, EGFR	250 uM	16 (2)	No	0.610
DNA damage	Celastrol	ROS-generating	20 uM	16 (2)	Yes	0.001
	Rotenone	ROS-generating	10 uM	16 (2)	No	0.149
	Cisplatin	alkylating agent	100 uM	16 (2)	No	0.065
	Camptothecin	Inhibits topoisomerase II	50 uM	16 (2)	No	0.059
	Teniposide	Inhibits topoisomerase ll	500 uM	16 (2)	Yes	< 0.001
	Cycloheximide	Ribosome inhibitor, prevents DNA damage	50 uM	16 (2)	Yes	< 0.001
Cell cycle delay	Colchicine	Destabilizes microtubules	2.5 uM	16 (2)	No	0.967
	Griseofulvin	Destabilizes microtubules	300 uM	16 (2)	No	0.675
	Taxol	Stabilizes microtubules	1 uM	16 (2)	No	0.774
	Apcin	APC/C inhibitor	300 uM	16 (2)	No	0.061
	TAME	APC inhibitor	20 mM	16 (2)	No	0.410
	Flavopiridol	CDK inhibitor	10 uM	16 (2)	No	0.360
	Roscovitine	CDK inhibitor	100 uM	16 (2)	No	0.175
	Bortezomib	Proteasome inhibitor	1 uM	16 (2)	Yes	0.005
	MG132	Proteasome inhibitor	50 uM	16 (2)	Yes	0.001
	Trametinib	MEK inhibitor	250 nM	16 (2)	Yes	0.002

NaBu is best known for its impact on chromatin structure and has been credited with suppressing HDACs 1–5 and 7–9, representing the entirety of class I and IIa HDAC enzymes (Ganai et al., 2016; Tandon et al., 2016). To determine whether maternal HDAC function affects *w*Ri-induced CI, an array of HDAC inhibitors was dose-optimized (Supplementary Table S6) and tested in the plate-based assay. The selected inhibitors included the class I and II HDAC inhibitors quisinostat (Arts et al., 2009) and trichostatin A, as well as the pan HDAC inhibitor vorinostat (SAHA) (Ganai et al., 2016; Tandon et al., 2016). The inhibitor CUDC-101, which targets class I and II HDAC as well as growth factor receptors, was also tested (Lai et al., 2010). Remarkably, none of these HDAC inhibitors exerted significant impact on CI hatch rates (Table 3) (Supplementary Tables S7, S8). To confirm that the fly stocks and assay parameters were still performing as expected, the effect of NaBu was retested in the plate-based format. The data confirmed that CI suppression by NaBu was still significant, with a *p*-value below 0.001 (Table 3) (Supplementary Figure S1 and Supplementary Tables S7–S9). Overall, these results do not support HDAC inhibition and associated chromatin remodeling as a generalized mechanism for CI suppression.

### Testing Modifiers of DNA Damage for Maternal CI Suppression Effects

NaBu has also been shown to promote DNA repair, in part by indirectly increasing acetylation of histone H4 (Smerdon et al., 1982; Williamson et al., 2012; Mao and Wyrick, 2016). To test whether maternal DNA repair processes affect *w*Ri-induced CI, an array of inhibitors was pursued. To activate the DNA repair response, agents that induce oxidative DNA damage were selected, specifically celastrol and rotenone (Sanders and Greenamyre, 2013; Xu et al., 2013; Moreira et al., 2019). The alkylating agent cisplatin, which also generates reactive oxygen, was included as well (Basu and Krishnamurthy, 2010; Podratz et al., 2011; Rezaee et al., 2013). Topoisomerase inhibitors were also used, including camptothecin, which prevents re-sealing of single stranded nicks by topoisomerase I, and teniposide, which prevents removal of topoisomerase II from DNA and induces degradation of the enzyme (Rowe et al., 1986; Hartmann and Lipp, 2006; Nitiss, 2009). As the ribosome inhibitor cycloheximide has been reported to prevent formation of single- and double-stranded DNA breaks (Yoshioka et al., 1987; Lorico et al., 1988), this drug was also tested.

Using optimized doses (Supplementary Table S6), the plate-based feeding assay indicated CI suppression for half of the treatment conditions used. A significant increase in hatch rate was observed for uninfected females exposed to celastrol, teniposide and cycloheximide, all associated with *p*-values of 0.001 or less (Table 3) (Supplementary Figure S1 and Supplementary Tables S7–S9). These data open a possible role for maternal processes that prevent and repair DNA damage in conferring CI suppression upon uninfected embryos.

### Testing the Impact of Cell-Cycle Timing on Maternal CI Suppression

Exposure to NaBu has been shown to slow cell cycle timing (D’Anna et al., 1980; Lallemand et al., 1996). To test the effect of maternal cell cycle timing on *w*Ri-induced CI, uninfected *D. simulans* were exposed to an array of complementary inhibitors. In attempt to slow the progression of mitosis by altering microtubule dynamics, the microtubule destabilizers colchicine and griseofulvin, as well as the microtubule stabilizer taxol were tested (Singh et al., 2008; Stanton et al., 2011). To slow anaphase onset and exit from mitosis, inhibitors of the anaphase promoting complex, apcin and TAME, were used (Zeng et al., 2010; Sackton et al., 2014). To inhibit the progress of mitosis and the cell cycle overall, the Cyclin dependent kinase inhibitors flavopiridol and roscovitine were used (Gray et al., 1999; Cicenas et al., 2015; Bailon-Moscoso et al., 2017), as well as the proteasome inhibitors bortezomib and MG132 (Goldberg, 2012; Rastogi and Mishra, 2012). To stall general re-entry into the cell cycle, the MAPKK (MEK) inhibitor trametinib was also used (Zeiser, 2014; Kurata et al., 2016).

After identifying appropriate doses (Supplementary Table S6), chemical manipulators of cell cycle timing were tested for CI suppression. The plate assay data indicated significantly increased CI hatch rates for bortezomib, MG132, and trametinib-fed females compared to control (*p*-value range: 0.001–0.005) (Table 3) (Supplementary Figure S1 and Supplementary Tables S7–S9). As cell cycle delays are a recognized consequence of DNA damage, resulting from checkpoint activation that allows damage repair (Chao et al., 2017), these data are consistent with a possible role for altered embryonic cell cycle timing in suppression of CI.

### Re-testing CI-Suppressing Compounds Against Transinfected *D. simulans*

If the compounds that suppress *w*Ri-induced CI act upon a network of conserved, Rescue-related maternal interactions, the effects would be expected to be applicable to other host-strain combinations. To this end, the chemicals identified above as hits were analyzed for suppression of *w*Mel-induced CI as well, using flies from a transinfected stock population (Poinsot et al., 1998). Specifically, NaBu, celastrol, cycloheximide, teniposide, bortezomib, MG132, trametinib, and the initially borderline hit acetic acid (Table 3) were retested in the plate assay format. The same dosing and procedures were used as above, with the only difference being that *w*Mel-infected males were used for CI induction.

The results indicated that NaBu, celastrol, and cycloheximide significantly elevated CI hatch rates for *w*Mel-induced CI (*p*-value range: 0.011–0.013) (Table 4) (Supplementary Figure S2 and Supplementary Tables S10–S12). By contrast, teniposide, bortezomib, and MG132 treatments exhibited borderline CI suppression effects (*p*-value range 0.041–0.047). Trametinib and acetic acid did not induce any significant effects (Table 4) (Supplementary Tables S10, S11). This outcome distinguishes cellular responses associated with certain DNA damage and/or cell cycle timing regulators as general contributors to maternal suppression of CI in *D. simulans.*

**TABLE 4:** Impact of chemical treatments on CI egg hatch rates for *w*Mel-infected *D. simulans.*

Effect tested	Chemical used	Reputed cellular effect	Dose used	# wells (plates)	Sig increase in hatch rate?	*p*-value
Diverse functions	NaBu	C4 short chain fatty acid. Affects HDACs, DNA damage repair, cell cycle	50 mM	16 (2)	Yes	0.011
Short chain fatty acids	Acetic acid	C2 short chain fatty acid	100 mM	16 (2)	No	0.408
DNA damage	Celastrol	ROS-generating	20 uM	16 (2)	Yes	0.013
	Cycloheximide	Ribosome inhibitor, prevents DNA damage	50 uM	16 (2)	Yes	0.013
	Teniposide	Inhibits topoisomerase ll	500 uM	16 (2)	Borderline	0.041
Cell cycle delay	Bortezomib	Proteasome inhibitor	1 uM	16 (2)	Borderline	0.047
	MG132	Proteasome inhibitor	50 uM	16 (2)	Borderline	0.047
	Trametinib	MEK inhibitor	250 nM	16 (2)	No	0.096

### Testing Combined Pathway Effects for Suppression of CI

To further test the extent to which suppression of CI under these treatments is due to a shared network of pathway functions, a dual chemical treatment strategy was pursued. We focused on hits that elicited the most robust CI suppression across both systems tested, namely: NaBu, celastrol, and cycloheximide. Additional treatment combinations included compounds that exerted more modest effects, namely: teniposide, bortezomib, and MG132. As for the single drug trials, dose response curves were carried out for all pairwise chemical treatments on uninfected *D. simulans* flies (Supplementary Table S13). Nearly all treatment combinations required reduced drug dosing, as compared to singly administered treatments (Supplementary Table S6). The cycloheximide/bortezomib combination was the only case in which original treatment doses for both compounds could be tolerated additively, without adverse effects on fecundity, egg hatch or larval development (Supplementary Table S13).

After uninfected *D. simulans* females were treated with dual drug combinations, their egg hatch rates were compared against females raised on control food in the plate assay format. To ensure that the results would be representative of natural CI, *w*Ri-infected males were used to induce CI in this series of experiments. The results indicated that the cycloheximide/bortezomib combination significantly increased the CI hatch rate as compared to the CI control (*p* = 0.006) (Table 5) (Supplementary Tables S14, S15). No other paired chemical treatments induced CI suppression (Table 5). It is possible that loss of CI suppression effects is attributable to reduced combinatorial doses of otherwise effective compounds. Regardless, the data indicate CI suppression by compounds that are traditionally associated with manipulation of protein synthesis and protein turnover.

**TABLE 5:** Impact of chemical combinations on CI egg hatch rates for *w*Ri-infected *D. simulans.*

Effect tested	Chemical used	Dose used	# wells (plates)	Significant increase in hatch rate?	*p*-value
Diverse functions/DNA damage	NaBu/Celastrol	25 mM/10 μM	16 (2)	No	0.119
Diverse functions/DNA damage	NaBu/Cycloheximide	25 mM/25 μM	16 (2)	No	0.817
Diverse functions/cell cycle delay	NaBu/MG132	25 mM/25 μM	16 (2)	No	0.201
DNA damage	Teniposide/Celastrol	250 μM/500 μM	16 (2)	No	0.287
DNA damage	Celastrol/Cycloheximide	10 μM/25 μM	16 (2)	No	0.565
DNA damage/cell cycle delay	Cycloheximide/Bortezomib	50 μM/1 μM	16 (2)	Yes	0.006
DNA damage/cell cycle delay	Teniposide/MG132	250 μM/25 μM	16 (2)	No	0.264

## Discussion

This study was designed to inform host capacity for modifying CI outcomes in the context of non-model organisms. A strong case is currently being made for involvement of *Wolbachia* Cif proteins in induction of CI and Rescue, based on analysis of *D. melanogaster* and yeast models (Beckmann et al., 2017, 2019b; LePage et al., 2017; Shropshire et al., 2018; Chen et al., 2019). This study, using natural and transinfected *D. simulans*, add to the complex biological underpinnings of embryonic lethality by indicating that maternal contributions to Rescue are also possible. Demonstrating CI suppression through chemical feeding of uninfected females, without invoking any effect or contribution by *Wolbachia*-supplied antitoxins, opens the possibility of a role for host contributions to Rescue. Fundamental to these questions is whether a core set of maternal mechanisms in insects can act to suppress CI across systems. Fortunately, the framework presented here enables broad investigation of diverse CI-Rescue systems in the future, including that of non-*Wolbachia* endosymbionts like *Cardinium* (Hunter et al., 2003; Penz et al., 2012; Mann et al., 2017; Takano et al., 2017; König et al., 2019; Doremus and Hunter, 2020).

Despite the reproducibility and statistical significance of CI-suppression effects by multiple drugs and drug combinations, the limitations of the current work are reflected by CI hatch rates which did not exceed three times that of CI-control hatch rates. The disparity between the 90%+ egg hatch frequencies of *Wolbachia-*induced Rescue and that of chemical CI suppression can be due to a variety of factors or experimental limitations. One important aspect could be the absence of *Wolbachia-*supplied Cif proteins. It is possible that maternal mechanisms act as a supplement to, or in coordination with Cif functions to a substantial extent. It also remains possible that CI-suppressing treatments mimic Cif effects, even in the absence of a usual role for host factors in Rescue. There are no known chemical treatments that will specifically mimic predicted Cif proteins at this time (Beckmann et al., 2017; Lindsey et al., 2018). Our attempt to alter broader functional networks of maternal proteins by use of multiple inhibitors was met with limited success, possibly due to dosage considerations, limited by systemic tolerances for dual treatments.

When interpreting the CI suppression data yielded by the current chemical screen, the technical limitations inherent to the method itself are important to consider. Whole body feedings lack the time and tissue-specific nuance afforded to *Wolbachia in vivo.* While dosing within the food was standardized for this study, it is not possible to control for local dosing to tissues/cells of the recipient organism. This is due to differences in ingestion, absorption, efflux, metabolism, and/or excretion rates, which are expected to vary in association with each cell type and each drug. A feeding assay also creates the possibility for side effects due to host microbiome impacts. Initial attempts to run this screen using standardized micro-injections were curtailed by observations that injected *D. simulans* flies stop laying eggs. Working with flies under anexic or gnotobiotic conditions presents its own set of complications (Koyle et al., 2016). For these reasons, seeing a response from this chemical screen is informative, whereas the lack of a response is not. Distinct from most drug screens, whose interpretations are limited by typically detrimental health effects on the test subject, the output of this screen leads to increased survival of otherwise ill-fated embryos.

This study was designed to address contributions of host chromatin remodeling, DNA damage repair and cell cycle timing impacts on CI suppression *in vivo*. Though little support was evident for chromatin remodeling in CI suppression, impacts on DNA integrity and cell cycle timing conferred significant increases in CI egg hatch. As such, our results at present do not readily distinguish between existing models of CI and Rescue, but instead opens consideration of networked models that may better reflect the cell biology of CI. DNA damage and cell cycle timing are well-known to be intrinsically connected, since DNA damage triggers a checkpoint mechanism that arrests the cell cycle (Alberts et al., 2015; Chao et al., 2017). Variation upon functions of the ubiquitin-proteasome system may further link these processes. DNA damage repair is facilitated by ubiquitination of histones and DNA repair pathway proteins, followed by their deubiquitination upon completion of the repair (Cohn and D’Andrea, 2008; Stadler and Richly, 2017; Uckelmann and Sixma, 2017). Ubiquitination and degradation of cyclins and other regulators is also fundamentally required for cell cycle progression (Bassermann et al., 2014; Alberts et al., 2015). The deubiquitylase and nuclease functions of Cif proteins converge upon these same processes (Beckmann and Fallon, 2013; Beckmann et al., 2017, 2019a,b; Lindsey et al., 2018; Chen et al., 2019), and expression studies in CI-inducing *Cardinium* have implicated the ubiquitin-proteasome system and DNA repair as well (Mann et al., 2017). The CI-suppressing compounds identified in this study reflect this continuum of function.

**FIGURE 5:**
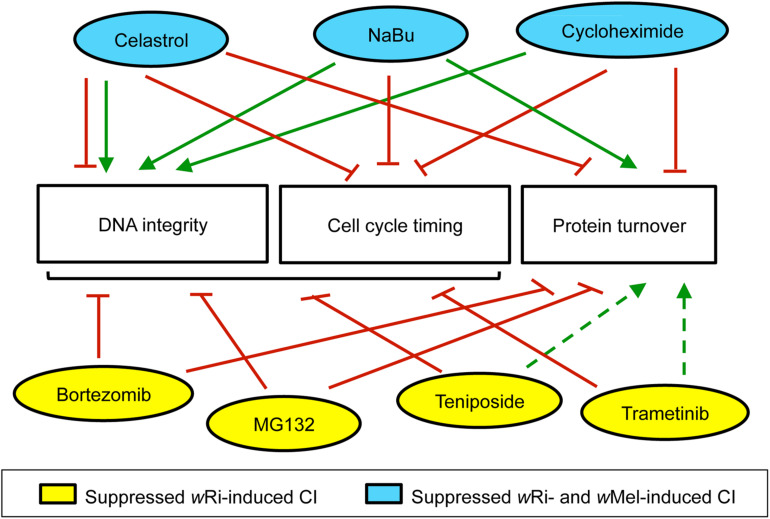
Summary of maternal impacts that significantly increased *D. simulans* CI hatch rates. This information is based upon available literature, summarized in Additional File S3. Green arrows: positive impact. Red lines: negative effect. Dotted lines: interpretation based upon partial datasets. Bracket: includes multiple categories.

One question raised by this work is what distinguished the compounds that affected egg hatch for both *w*Ri and *w*Mel-induced CI, from those that affected *w*Ri-induced CI only (Figure 5). As *w*Ri and *w*Mel encode different types of Cif proteins, Cin versus Cid, respectively (Beckmann and Fallon, 2013), it would be reasonable for CI associated with different strains to respond differently to the same CI-suppressing compounds. In general, this study found that “hit” compounds that suppressed *w*Ri-induced CI exerted overall weaker effects on *w*Mel-induced CI. It is not clear whether differences in Cif proteins are responsible, as other circumstantial explanations could also cause this outcome. Future tests of CI-suppressing compounds across more host-strain combinations will be needed to make a stronger correlative statement addressing this point.

Another perspective for interpretation regarding the cellular impacts of the compounds themselves. Is there anything distinct about the activity profile of compounds that suppress both *w*Ri and *w*Mel-induced CI? The use of well-known compounds in this study provides access to vast literature for interpreting reproducible CI suppression effects. Past studies of mammalian systems and cell lines provide a patchwork of information that informs effects on DNA integrity, cell cycle timing and protein turnover. According to existing literature, the compounds identified here as CI-suppressing agents act as consistent suppressors of cell cycle timing, but exert variable impacts on protein turnover, depending upon the compound (Additional File S3). Although neither of those functional profiles aligns with the strain-specific differences observed in CI egg hatch outcomes thus far (Figure 5).

Clearer associations are evident between existing literature and CI suppression outcomes in this study from the perspective of DNA integrity. The four compounds that suppress *w*Ri-induced CI are known inducers of DNA damage, whereas the three compounds that suppress both *w*Ri- and *w*Mel-induced CI, reportedly support DNA integrity (Figure 5) (Additional File S3). Though celastrol can exert detrimental impacts on DNA (Han et al., 2018; Wang et al., 2020), it has also been shown to suppress radiation-induced damage (Xu et al., 2013; Moreira et al., 2019). Cycloheximide treatments prevent formation of single- and double-strand DNA breaks (Yoshioka et al., 1987; Lorico et al., 1988). NaBu protects DNA integrity by up-regulating antioxidant pathways and by facilitating DNA repair (Smerdon et al., 1982; Mao and Wyrick, 2016; El-Shorbagy, 2017). This suggests that DNA integrity is a dynamic, focal aspect of CI suppression with respect to different *Wolbachia* strains in *D. simulans*. Future analyses of CI and Rescue, from the perspective of both host and microbe promise to be informative in elucidating the molecular basis of this ecologically relevant mechanism.

## Data Availability Statement

The datasets GENERATED for this study can be found in NCBI SRA https://www.ncbi.nlm.nih.gov/bioproject/PRJNA663645.

## Author Contributions

AJMZM, AAS, SS, SC, and LS supervised the experiments. AJMZM, AAS, JGA, SS, AF, JH, and SC conducted the experiments. AJMZM, AAS, JH, SC, and LS wrote the manuscript. All authors designed the experiments and reviewed the manuscript.

## Conflict of Interest

The authors declare that the research was conducted in the absence of any commercial or financial relationships that could be construed as a potential conflict of interest.

## Footnotes

1 https://bdsc.indiana.edu/information/recipes/bloomfood.html

## Supplementary Material

Supplementary Figure 1 Sub-sampling analyses, in tests for chemical suppression of *w*Ri-induced CI. The likelihood of satisfying set *p*-values is shown for “hit” compounds identified by chemical screening. Compound treatments are color coded as indicated at the bottom of the figure. For NaBu, the data reached 100% satisfaction of the cutoff *p* < 0.05 at 12 wells, and *p* < 0.01 at 15 wells. For Teniposide, the data reached 100% satisfaction of the cutoff *p* < 0.05 at 11 wells, and *p* < 0.01 at 14 wells. All other conditions showed a 95%+ likelihood of satisfying *p* < 0.05, and an 80%+ likelihood of satisfying *p* < 0.01, at *n* = 16 wells.

Supplementary Figure 2 Sub-sampling analyses, in tests for chemical suppression of *w*Mel-induced CI. The likelihood of satisfying set *p*-values is shown for “hit” compounds identified by chemical screening. Compound treatments are color coded as indicated at the bottom of the figure. The data showed a 72–82% likelihood of satisfying *p* < 0.05, and a 47–72% likelihood of satisfying *p* < 0.01, at *n* = 16 wells.
