# Supplementary material for: A Role for Maternal Factors in Suppressing Cytoplasmic Incompatibility
Source: Front Microbiol. 2020 Nov 9;11:576844. doi: 10.3389/fmicb.2020.576844 (PMC7680759; doi:10.3389/fmicb.2020.576844)
Supplement: Supplementary Figure 1 — Sub-sampling analyses, in tests for chemical suppression of wRi-induced CI. The likelihood of satisfying set p-values is shown for “hit” compounds identified by chemical screening. Compound treatments are color coded as indicated at the bottom of the figure. For NaBu, the data reached 100% satisfaction of the cutoff p < 0.05 at 12 wells, and p < 0.01 at 15 wells. For Teniposide, the data reached 100% satisfaction of the cutoff p < 0.05 at 11 wells, and p < 0.01 at 14 wells. All other conditions showed a 95%+ likelihood of satisfying p < 0.05, and an 80%+ likelihood of satisfying p < 0.01, at n = 16 wells. [file Data_Sheet_1.zip › MomtazEtAlSupplementaryMaterial/MomtazEtAl_AdditionalFileS3.docx]

**Drug type**

Celastrol

NaBu

Cycloheximide

Teniposide

Bortezomib

and MG132

Trametinib

**DNA integrity**

Shown to cause DNA damage in some studies [1, 2]

Prevents DNA damage in certain conditions [3, 4]

__________________

Protects/prevents DNA damage [5, 6], in part by via antioxidant proteins [7] and in part via histone H4 acetylation [8, 9]

__________________

Prevents formation of single-stranded [10] and double-stranded [11] DNA breaks

__________________

Primarily creates single-stranded DNA breaks [12]

__________________

Inhibit DNA damage repair by acting on ATM kinase and other DNA repair pathway factors [13]

__________________

Inhibits DNA damage repair under certain circumstances [14], possibly by acting on ATM kinase [15]

_________________

**Cell cycle timing**

Induces cell cycle arrest [16, 17]

Down-regulates Cyclin D, which normally drives re-entry into G1 [18]

__________________

Inhibits cell cycle in a concentration-dependent manner [19], possibly by suppressing Cyclin D expression [20]

__________________

Slows the cell cycle, primarily in G1 and S phase [21, 22]

__________________

Inhibits cell cycle in a concentration-dependent manner [23]

__________________

Induce cell cycle arrest at G2/M [24]

__________________

MEK inhibitor, drives cell cycle arrest at G0 and G1 [25, 26]

__________________

**Protein turnover**

Inhibits proteasome activity [27]

Enhances effect of Bortezomib and MG132 [28, 29]

__________________

Stimulates proteasome activity [30]

__________________

Inhibits production of ubiquitin subunits [31]

__________________

**Induces proteasomal degradation of certain targets [32]

__________________

Directly inhibit proteasome activity [33]

__________________

**Induces proteasomal degradation of certain targets [34]

__________________

** *narrowly tested*

**References:**

1. Wang, H., et al., *Celastrol Alleviates Gamma Irradiation-Induced Damage by Modulating Diverse Inflammatory Mediators.* Int J Mol Sci, 2020. **21**(3).

2. Han, X.B., et al., *Protective effects of celastrol against γ irradiation-induced oxidative stress in human umbilical vein endothelial cells.* Exp Ther Med, 2018. **16**(2): p. 685-694.

3. Moreira, H., et al., *Prooxidative Activity of Celastrol Induces Apoptosis, DNA Damage, and Cell Cycle Arrest in Drug-Resistant Human Colon Cancer Cells.* Oxid Med Cell Longev, 2019. **2019**: p. 6793957.

4. Xu, Z., et al., *Celastrol induced DNA damage, cell cycle arrest, and apoptosis in human rheumatoid fibroblast-like synovial cells.* Am J Chin Med, 2013. **41**(3): p. 615-28.

5. Rosignoli, P., et al., *Protective activity of butyrate on hydrogen peroxide-induced DNA damage in isolated human colonocytes and HT29 tumour cells.* Carcinogenesis, 2001. **22**(10): p. 1675-80.

6. Li, L., et al., *Histone deacetylase inhibitor sodium butyrate suppresses DNA double strand break repair induced by etoposide more effectively in MCF-7 cells than in HEK293 cells.* BMC Biochem, 2015. **16**: p. 2.

7. El-Shorbagy, H.M., *Potential anti-genotoxic effect of sodium butyrate to modulate induction of DNA damage by tamoxifen citrate in rat bone marrow cells.* Cytotechnology, 2017. **69**(1): p. 89-102.

8. Mao, P. and J.J. Wyrick, *Emerging roles for histone modifications in DNA excision repair.* FEMS Yeast Res, 2016. **16**(7).

9. Smerdon, M.J., et al., *Sodium butyrate stimulates DNA repair in UV-irradiated normal and xeroderma pigmentosum human fibroblasts.* J Biol Chem, 1982. **257**(22): p. 13441-7.

10. Lorico, A., et al., *Accumulation of DNA strand breaks in cells exposed to methotrexate or N10-propargyl-5,8-dideazafolic acid.* Cancer Res, 1988. **48**(8): p. 2036-41.

11. Yoshioka, A., et al., *Deoxyribonucleoside triphosphate imbalance. 5-Fluorodeoxyuridine-induced DNA double strand breaks in mouse FM3A cells and the mechanism of cell death.* J Biol Chem, 1987. **262**(17): p. 8235-41.

12. Long, B.H., S.T. Musial, and M.G. Brattain, *Single- and double-strand DNA breakage and repair in human lung adenocarcinoma cells exposed to etoposide and teniposide.* Cancer Res, 1985. **45**(7): p. 3106-12.

13. Jacquemont, C. and T. Taniguchi, *Proteasome function is required for DNA damage response and fanconi anemia pathway activation.* Cancer Res, 2007. **67**(15): p. 7395-405.

14. Maertens, O., et al., *MAPK Pathway Suppression Unmasks Latent DNA Repair Defects and Confers a Chemical Synthetic Vulnerability in.* Cancer Discov, 2019. **9**(4): p. 526-545.

15. Smida, M., et al., *MEK inhibitors block growth of lung tumours with mutations in ataxia-telangiectasia mutated.* Nat Commun, 2016. **7**: p. 13701.

16. Kashyap, D., et al., *Molecular targets of celastrol in cancer: Recent trends and advancements.* Crit Rev Oncol Hematol, 2018. **128**: p. 70-81.

17. Song, X., Y. Zhang, and E. Dai, *Therapeutic targets of thunder god vine (Tripterygium wilfordii hook) in rheumatoid arthritis (Review).* Mol Med Rep, 2020. **21**(6): p. 2303-2310.

18. Kannaiyan, R., et al., *Celastrol inhibits proliferation and induces chemosensitization through down-regulation of NF-κB and STAT3 regulated gene products in multiple myeloma cells.* Br J Pharmacol, 2011. **164**(5): p. 1506-21.

19. D'Anna, J.A., R.A. Tobey, and L.R. Gurley, *Concentration-dependent effects of sodium butyrate in Chinese hamster cells: cell-cycle progression, inner-histone acetylation, histone H1 dephosphorylation, and induction of an H1-like protein.* Biochemistry, 1980. **19**(12): p. 2656-71.

20. Lallemand, F., et al., *Direct inhibition of the expression of cyclin D1 gene by sodium butyrate.* Biochem Biophys Res Commun, 1996. **229**(1): p. 163-9.

21. Liu, X., et al., *Induction of cell cycle arrest at G1 and S phases and cAMP-dependent differentiation in C6 glioma by low concentration of cycloheximide.* BMC Cancer, 2010. **10**: p. 684.

22. Polymenis, M. and R. Aramayo, *Translate to divide: сontrol of the cell cycle by protein synthesis.* Microb Cell, 2015. **2**(4): p. 94-104.

23. Del Bino, G. and Z. Darzynkiewicz, *Camptothecin, teniposide, or 4'-(9-acridinylamino)-3-methanesulfon-m-anisidide, but not mitoxantrone or doxorubicin, induces degradation of nuclear DNA in the S phase of HL-60 cells.* Cancer Res, 1991. **51**(4): p. 1165-9.

24. Rastogi, N. and D.P. Mishra, *Therapeutic targeting of cancer cell cycle using proteasome inhibitors.* Cell Div, 2012. **7**(1): p. 26.

25. Kurata, K., et al., *Growth arrest by activated BRAF and MEK inhibition in human anaplastic thyroid cancer cells.* Int J Oncol, 2016. **49**(6): p. 2303-2308.

26. Zeiser, R., *Trametinib.* Recent Results Cancer Res, 2014. **201**: p. 241-8.

27. Yang, H., et al., *Celastrol, a triterpene extracted from the Chinese "Thunder of God Vine," is a potent proteasome inhibitor and suppresses human prostate cancer growth in nude mice.* Cancer Res, 2006. **66**(9): p. 4758-65.

28. Walcott, S.E. and J.J. Heikkila, *Celastrol can inhibit proteasome activity and upregulate the expression of heat shock protein genes, hsp30 and hsp70, in Xenopus laevis A6 cells.* Comp Biochem Physiol A Mol Integr Physiol, 2010. **156**(2): p. 285-93.

29. Shanmugam, M.K., et al., *Celastrol Attenuates the Invasion and Migration and Augments the Anticancer Effects of Bortezomib in a Xenograft Mouse Model of Multiple Myeloma.* Front Pharmacol, 2018. **9**: p. 365.

30. Giuliano, M., et al., *The apoptotic effects and synergistic interaction of sodium butyrate and MG132 in human retinoblastoma Y79 cells.* Cancer Res, 1999. **59**(21): p. 5586-95.

31. Hanna, J., D.S. Leggett, and D. Finley, *Ubiquitin depletion as a key mediator of toxicity by translational inhibitors.* Mol Cell Biol, 2003. **23**(24): p. 9251-61.

32. Yusenko, M., A. Jakobs, and K.H. Klempnauer, *A novel cell-based screening assay for small-molecule MYB inhibitors identifies podophyllotoxins teniposide and etoposide as inhibitors of MYB activity.* Sci Rep, 2018. **8**(1): p. 13159.

33. Berkers, C.R., et al., *Activity probe for in vivo profiling of the specificity of proteasome inhibitor bortezomib.* Nat Methods, 2005. **2**(5): p. 357-62.

34. Lin, L., et al., *Trametinib potentiates TRAIL-induced apoptosis via FBW7-dependent Mcl-1 degradation in colorectal cancer cells.* J Cell Mol Med, 2020.
