# Supplementary figures and images for: A Role for Maternal Factors in Suppressing Cytoplasmic Incompatibility
Source: Front Microbiol. 2020 Nov 9;11:576844. doi: 10.3389/fmicb.2020.576844 (PMC7680759; doi:10.3389/fmicb.2020.576844)

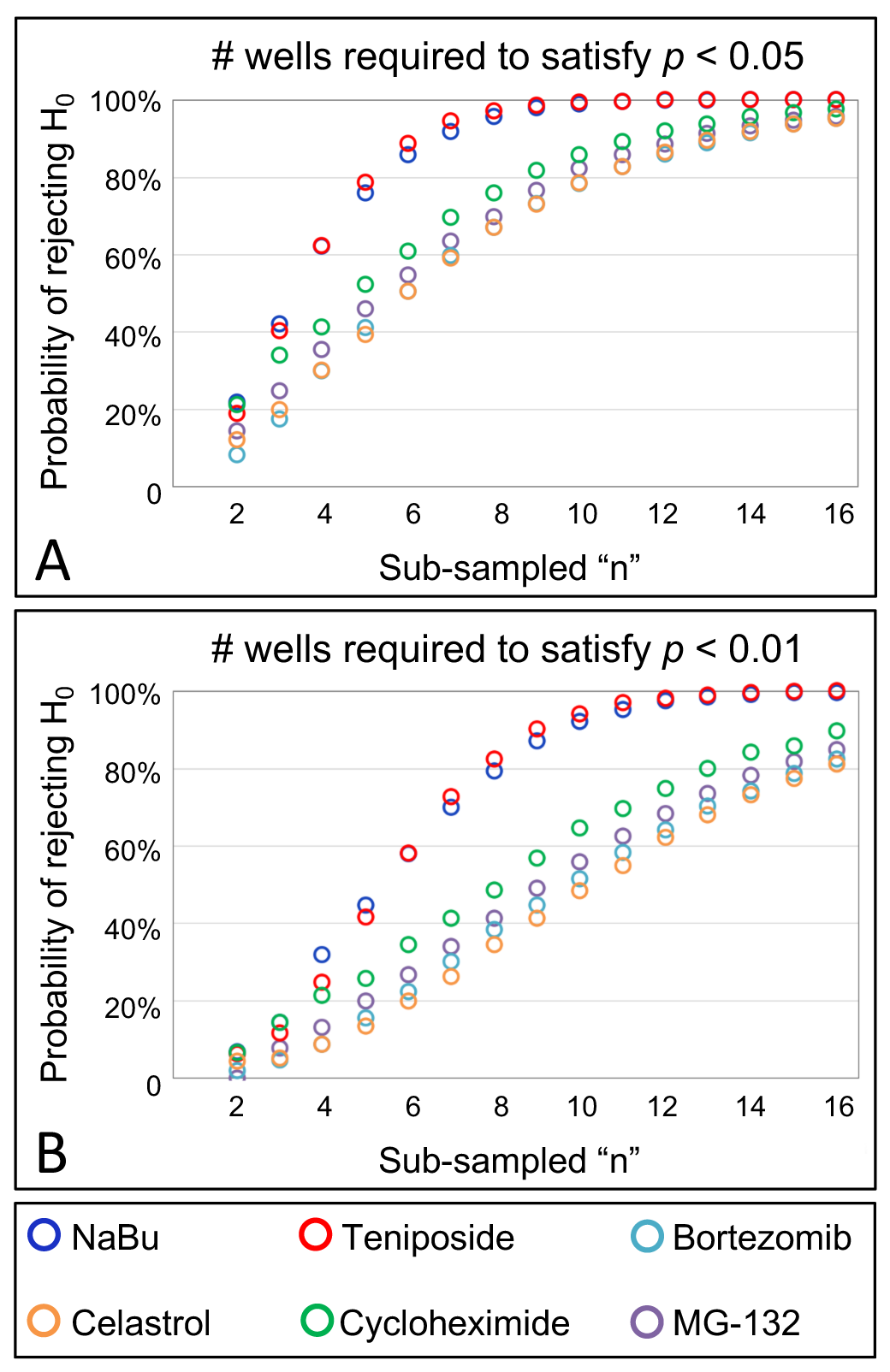

Supplement: Supplementary Figure 1 — Sub-sampling analyses, in tests for chemical suppression of wRi-induced CI. The likelihood of satisfying set p-values is shown for “hit” compounds identified by chemical screening. Compound treatments are color coded as indicated at the bottom of the figure. For NaBu, the data reached 100% satisfaction of the cutoff p < 0.05 at 12 wells, and p < 0.01 at 15 wells. For Teniposide, the data reached 100% satisfaction of the cutoff p < 0.05 at 11 wells, and p < 0.01 at 14 wells. All other conditions showed a 95%+ likelihood of satisfying p < 0.05, and an 80%+ likelihood of satisfying p < 0.01, at n = 16 wells. [file Data_Sheet_1.zip › MomtazEtAlSupplementaryMaterial/MomtazEtAl_SupplementaryFigureS1.tif]

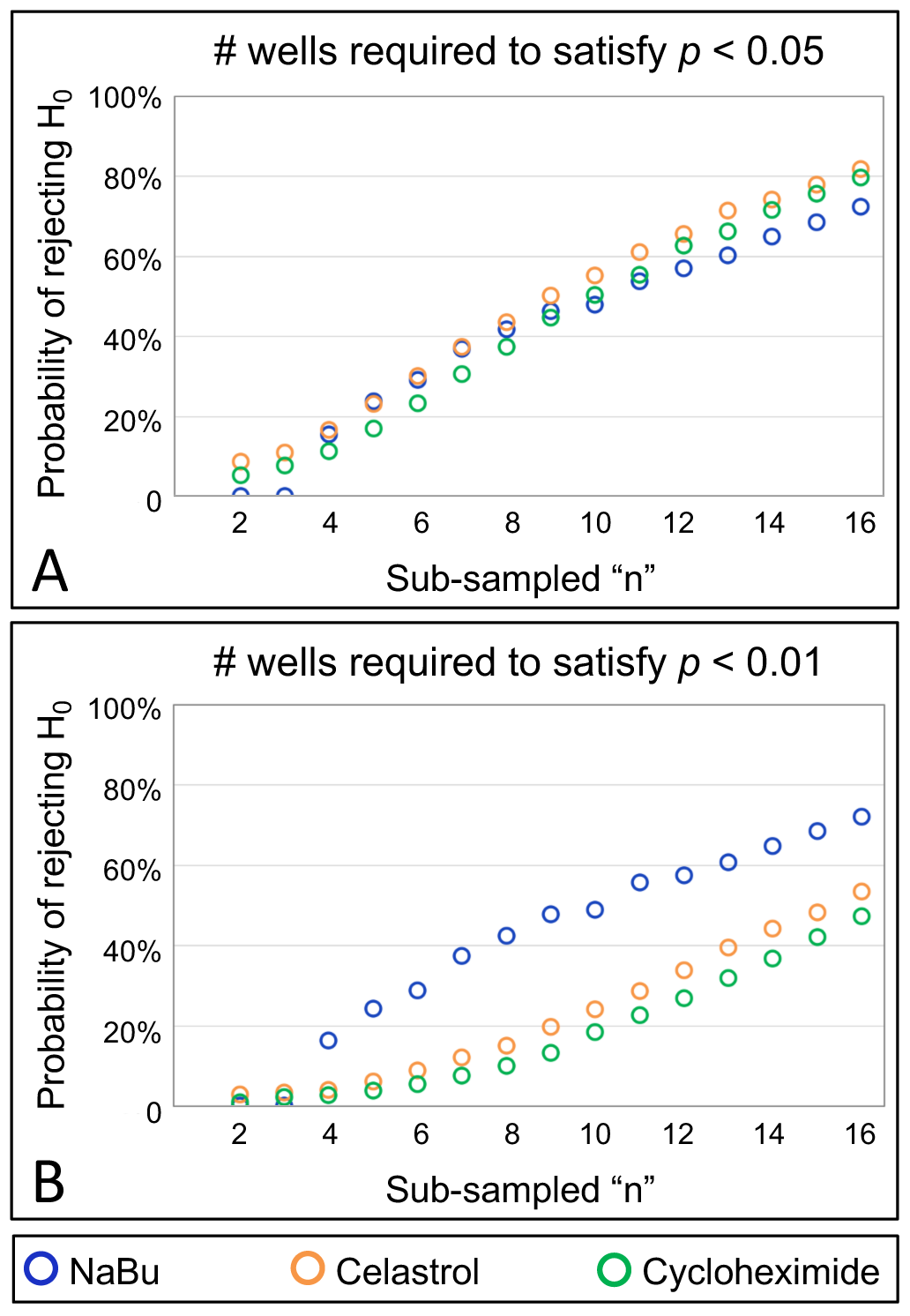

Supplement: Supplementary Figure 1 — Sub-sampling analyses, in tests for chemical suppression of wRi-induced CI. The likelihood of satisfying set p-values is shown for “hit” compounds identified by chemical screening. Compound treatments are color coded as indicated at the bottom of the figure. For NaBu, the data reached 100% satisfaction of the cutoff p < 0.05 at 12 wells, and p < 0.01 at 15 wells. For Teniposide, the data reached 100% satisfaction of the cutoff p < 0.05 at 11 wells, and p < 0.01 at 14 wells. All other conditions showed a 95%+ likelihood of satisfying p < 0.05, and an 80%+ likelihood of satisfying p < 0.01, at n = 16 wells. [file Data_Sheet_1.zip › MomtazEtAlSupplementaryMaterial/MomtazEtAl_SupplementaryFigureS2.tif]
